# The Role and Mandate of Nurses in Disasters: A Delphi Study in German‐Speaking Countries

**DOI:** 10.1111/inr.70167

**Published:** 2026-02-26

**Authors:** Michael Köhler, Julia Ballmann, Laura Glorius, Michael Ewers

**Affiliations:** ^1^ Charité – Universitätsmedizin Berlin, corporate member of Freie Universität Berlin and Humboldt‐Universität zu Berlin Institute of Health and Nursing Science Berlin Germany

**Keywords:** Delphi technique, disaster nursing, disaster planning, emergency responders, leadership, nurses’ role

## Abstract

**Background:**

Given the global increase in disasters, nurses play a critical role in ensuring the protection of individual and public health even under adverse circumstances. However, in Germany, Austria, and Switzerland, the role and mandate of nurses in disasters are unclear, which can lead to uncertainty among disaster responders, decision‐makers, and nurses themselves.

**Aim:**

The RAP^KAT^ study (The Role and Mandate of Nurses in Emergencies, Crises, and Disasters) aimed to examine the role and mandate of nurses in disasters from the perspective of experts across three German‐speaking countries.

**Methods:**

A modified multi‐stage Delphi design was conducted, using the DELPHISTAR checklist to report on this study. Following an initial round‐table symposium with experts from the field of nursing, three increasingly standardized online surveys were conducted. The panel of participants was gradually expanded to include experts from related fields and professions.

**Results:**

Participants from German‐speaking countries strongly agreed on the need to clearly define and codify the role and mandate of nurses in disaster situations. Experts, particularly nurses, called for the inclusion of disaster nurses in leadership and decision‐making bodies. However, participants also noted that disaster nursing competencies in these countries are not yet sufficiently developed to meet the expected standards.

**Conclusion:**

To ensure that nurses can respond appropriately to disasters, their role and mandate must be defined, codified, and systematically implemented. Additionally, disaster nursing competencies should be systematically developed through education.

**Implications for nursing and nursing policy:**

The responsibility for defining the role and mandate of nurses lies with the profession itself. Nurses must reach a consensus with policymakers on these definitions and ensure that they are codified, allowing healthcare systems to implement them effectively.

## Introduction

The role of nurses in disasters has gained increasing international recognition in recent decades (Fletcher et al. 2022), particularly in disaster‐prone regions such as the Asia Pacific (Bonito 2017). This growing acknowledgement reflects the rising global risks – from natural hazards and pandemics to geopolitical instability (WEF 2024) – that pose significant challenges to public health systems (Burkle 2019) and urgently demand effective healthcare responses.

The World Health Organization (WHO) and the International Council of Nurses (ICN) have long emphasized the essential role of nurses in disaster preparedness, response, and recovery (ICN 2019; WHO and ICN 2009), calling for clear role definitions, stronger leadership involvement, and systematic integration of nurses into strategic disaster planning and decision‐making structures (NASEM 2021). In response to changing global requirements, various institutions have rethought and redefined the nursing profession. For instance, in response to the COVID‐19 pandemic experience, the Royal College of Nursing has introduced a new definition of nursing as a safety‐critical profession (RCN 2023). The ICN has recently presented a new definition of nursing and nurses that explicitly refers to the role and responsibilities of this profession in the context of adversity. They see nurses at the front line, responding “*to disasters, conflicts and emergencies, demonstrating courage, dedication, adaptability and commitment to the health of individuals, communities and the environment*” (White et al. 2025, p. 12). Several years earlier, the ICN had defined and published the core competencies that nurses at all levels should possess before, during, and after disaster situations (ICN 2019, 2022).

In fact, as the largest group of healthcare professionals, nurses already play an important role in the context of disasters (NASEM 2021). They perform a wide range of tasks and functions in all phases of the disaster management cycle (Firouzkouhi et al. 2021; Fletcher et al. 2022). Frequently acting as first responders, nurses are key to safeguarding and maintaining healthcare even under extreme conditions. However, the scope of nursing practice goes beyond the provision of clinical care, encompassing roles such as education, coordination, and leadership. The COVID‐19 pandemic has brought the importance of nurses into sharper focus (Fawaz et al. 2020; Kang et al. 2024). Nevertheless, the strategic relevance has already been highlighted in international literature for a variety of disaster scenarios, ranging from natural hazards and technical disruptions to complex emergencies (e.g., Deeny and Davies 2018; Su et al. 2022). Public Health Nurses in particular are often valued for their community‐focused expertise in prevention, assessment, and coordination (Springer 2018).

Despite growing international engagement, there are also concerns that the capacity of nurses to respond to disasters is not fully considered (NASEM 2021). Moreover, nurses themselves often report uncertainty about their role and mandate (Atia et al. 2024) and frequently feel inadequately prepared for the demands they face before, during, and after disasters (Labrague et al. 2018). Challenges include not only clinical and organizational responsibilities, but also ethical and legal dilemmas, leadership expectations, coordination tasks, and psychological preparedness (Al Thobaity 2024). This may cause uncertainty among disaster response teams, decision‐makers, and nurses themselves, hindering an appropriate response to crises and disasters.

These observations may be particularly relevant for the German‐speaking countries – Germany, Austria, and Switzerland – for several reasons. These countries and their healthcare systems have historically been less exposed to major disasters, resulting in limited empirical experience and virtually no established body of research on nursing roles in such contexts. Only recently – partly in response to changing global threats – efforts have emerged to establish coherent disaster preparedness systems and corresponding policy frameworks (e.g., BMI 2022), including the clarification of healthcare professionals’ roles. However, these initiatives remain largely unimplemented, underlining a persistent gap between policy guidance and intended implementation (Thiebes et al. 2022). Not least, the nursing profession in this region is still confronted with structural disadvantages due to traditionally non‐academic education paths, delayed professionalization, and limited recognition within health policy and health care structures. This observation might be especially true for Germany, which is evident from the fact that the current guidelines on emergency and disaster medicine issued by the German Society for Anaesthesiology and Intensive Care Medicine (DGAI 2023) only classify nurses as spontaneous first responders, on a similar level to other citizens without qualifications. The potential contributions of the profession to civil protection and the maintenance of healthcare before, during, and after disasters were not given consideration.

## Study Aim

Against this background, we conducted a self‐funded research project on *The Role and Mandate of Nurses in Emergencies, Crises, and Disasters* (RAP^KAT^), which lasted from July 2023 to June 2025. The central component of RAP^KAT^ is the modified multi‐stage Delphi study reported here. In this project, we asked how the role and mandate of nurses in crises and disasters are currently perceived in the German‐speaking countries and what is needed to strengthen their contribution to disaster and crisis management within health and civil protection systems.

RAP^KAT^ aimed to initiate a structured professional and policy‐oriented discourse on the role of nurses in disaster contexts, foster expert consensus on key issues, and inform the development of future nursing policy, guidelines, and recommendations relevant to stakeholders and decision‐makers. The results of this study should ultimately help to improve the contribution of nurses to resilient healthcare systems and secure public health in crises and disasters in the respective countries and beyond.

The focus on German‐speaking countries (Germany, Austria, and Switzerland) was chosen deliberately. Beyond a shared language, these countries exhibit comparable professionalization trajectories in nursing, including a historically delayed academic development, limited formal mandates, and ongoing debates about professional autonomy, leadership, and the role of nurses in crisis and disaster contexts. This combination allows for the consideration of similarities as well as differences within a comparable linguistic and cultural context, while avoiding the methodological challenges associated with multilingual Delphi processes. Rather than comparing language regions per se, insights should be gained that are relevant to health systems facing similar professional and policy challenges.

## Methods

### Research Design

We conducted a modified Delphi study to gather expert judgements on the research topic. This methodology is particularly well‐suited for examining complex and under‐researched topics, initiating an opinion‐forming process among respondents with different social and professional backgrounds and, as a result, stimulating much‐needed social consensus‐building processes (Cuhls 2023). This study was reported on the DELPHISTAR checklist (Niederberger et al. 2024) in accordance with the standards established by the EQUATOR Network.

A team of health and nursing scientists conducted the Delphi study using a multi‐stage, hybrid approach (Landeta et al. 2011), combining the strengths of various techniques to effectively achieve the study's objectives. The number of rounds was defined in advance as four. The process began with a two‐day in‐person round‐table symposium on November 30 and December 1, 2023, in Berlin, Germany. In this first round, carefully selected experts from Austria, Germany, and Switzerland with a background in nursing, nursing policy, or disaster management were involved, though not all of whom were necessarily licensed nurses themselves. This initial phase was targeted to explore preliminary perspectives and identify potential strategies for clarifying nurses’ role and mandate in disaster contexts. Subsequently, we conducted three successive and increasingly standardized rounds of online surveys between February and August 2024, designed to iteratively refine and consolidate key statements. To broaden the perspective, the participant group was expanded in the final two survey rounds to include key stakeholders from civil protection, disaster response, humanitarian aid, and health policy. A summary of the Delphi study is illustrated in Table 1.

**TABLE 1:** Design of the multi‐stage Delphi Study.

	Initial stage	Second stage	Third stage	Fourth stage
Methods	Two‐day in‐person Round Table Symposium	Increasingly standardized online surveys		
Participants	Experts from nursing, politics, the armed forces, disaster relief organizations, and nursing education		Gradually expanded to include experts from related fields and professions	
Objective	Identification of positions and assumptions on nurses’ roles and mandates in disasters	Summary, weighting, and consolidation of the round table's topics	Refining and quantifying the consensus points with a broader expert perspective	

### Sample

Given the exploratory and interdisciplinary nature of the topic, we have applied a broad understanding of expertise when identifying the experts for the Delphi study, knowing very well that the selection of panel members is one of the most important aspects of this research method (Niederberger et al. 2023). Participants were considered experts if they met one or more of the following criteria: (1) professional role or experience in nursing and health policy, health security, disaster management or civil protection; (2) institutional affiliation with relevant organizations or authorities in this field; (3) documented thematic engagement with crisis and disaster management, or nursing policy at national or regional level. A purposeful sampling was conducted based on criteria such as the experts’ current roles and institutional context. This approach allowed for the inclusion of participants with diverse perspectives relevant to the study's objectives without narrowing the focus too much, for example, by requiring proven scientific or political expertise in the respective field.

The 28 participants of the round‐table symposium were selected from key stakeholder groups associated with the nursing profession across Germany, Austria, and Switzerland. Of the 26 who participated in the first online survey, five were excluded due to incomplete responses, leaving 21 valid data sets. Nineteen respondents stated that they were licensed in a regulated health profession, though only four were employed in healthcare or nursing care facilities, and others worked in fields such as education, disaster relief organizations, politics, and the armed forces.

For the expanded expert panel in the subsequent Delphi rounds (stages 3 and 4), individuals without a direct nursing background but with relevance to the study topic were identified. These included professionals from various related sectors and institutions in Germany: health policy actors, including the chairpersons and deputy chairpersons of health committees at the federal state level, representatives of interior affairs committees responsible for disaster preparedness, and chairpersons or subject matter experts (in nursing, health, or medicine) from national aid organizations such as the German Red Cross. Additionally, directors or designated representatives of relevant research and educational institutions in the fields of health, nursing, disaster relief, and civil protection were involved. The invited expert panel also included leaders or appointed members of professional associations for emergency and disaster medicine, and other relevant organizations (e.g., fire department association) and agencies, as well as individuals recognized for their contributions to research in this field. Equivalent organizations and representatives from Austria and Switzerland were also identified and included.

Together with the participants of the round‐table symposium, respectively, the first online survey, a total of 229 individuals were invited to take part in the third and fourth stages of the Delphi process. After excluding unusable data sets, 74 questionnaires from the third round and 64 from the fourth round were included in the final analysis – corresponding to response rates of 32% and 28%, respectively.

As shown in Table 2, more than 60% of participants in the second online survey were licensed nurses. This proportion decreased slightly to just under half in the final survey round. There were only minor variations in the distribution of participants’ occupational fields between the two surveys, with the majority continuing to work in the healthcare sector. Accordingly, the average self‐assessed level of expertise in the field of nursing was relatively high across both survey rounds. In contrast, expertise in civil protection was rated lower, though with notable deviation. A similar pattern applied to prior engagement with the topic, which varied considerably among participants.

**TABLE 2:** Sample characteristics of the third and fourth Delphi stages.

Characteristics	Third Delphi stage	Fourth Delphi stage
	(Online survey round 2)	(Online survey round 3)
Sample [% (*n*)]	100% (74)	100% (64)
Professional background [% (*n*)]		
Nurse	62.1 (46)	46.9 (30)
Other than a nurse	37.9 (28)	53.1 (34)
Professional field of activity (predominantly) [% (*n*)]		
Health and nursing care	28.4 (21)	21.9 (14)
Security sector (emergency services, military)	5.2 (4)	7.8 (5)
Disaster management	6.8 (5)	6.3 (4)
Politics/public administration/associations	14.9 (11)	18.8 (12)
Education/teaching	17.6 (13)	14.1 (9)
Science/research	14.9 (11)	15.6 (10)
Other field of activity	12.2 (9)	12.5 (8)
Self‐assessment of expertise in the field of… [M (SD)]^a^		
Nursing	81.0 (23.2)	75.6 (27.4)
Civil protection	66.3 (29.9)	68.0 (27.8)
Extent of prior engagement with the topic before survey participation [M (SD)]^b^	4.55 (1.37)	4.81 (1.19)

^a^ 0 = No expertise at all; 100 = high level of expertise.

^b^ 1 = Not at all; 6 = Very often.

### Data Collection

Given the limited attention paid to the role and mandate of nurses in disaster contexts in German‐speaking countries, the in‐person phase of the Delphi study was deliberately designed to include various inputs. This included a live presentation on site by one of the authors (ME) on international developments in emergency and disaster management, as well as various contributions from internationally recognized experts in the field of disaster nursing in the form of video recordings. This was followed by several guided discussion rounds with all experts and intensive parallel breakout sessions in smaller groups.

The first breakout session focused on different care settings, including emergency and acute care, long‐term care, primary and community care, and the broader context of civil protection, disaster response, and humanitarian aid. The second breakout session addressed education and professional development of nurses in disaster contexts, including the integration of disaster topics into basic nursing curricula, the development of continuing and advanced training programmes, role specialization and professionalization, and the competencies required for disaster nursing. The breakout sessions were guided by a small set of predefined lead questions to ensure comparability across groups. In the care setting groups, guiding questions focused on the current role and mandate of nurses in the respective setting. In the breakout sessions on education and professional development, discussions were guided by questions addressing what is necessary to establish nursing as a safety‐critical profession in disaster contexts. Across all groups, discussions followed a common structure addressing the current situation, identifying needs and pathways for implementation. The results of the discussion rounds were documented in written minutes by members of the extended research team.

Based on these discussions during the initial face‐to‐face phase of the Delphi study, a partially standardized questionnaire was developed for the first online survey by members of the research team. It consisted of four thematic sections: (1) the relevance of the topic and the need for a clearly defined role and mandate for nurses in emergencies, crises, and disasters; (2) positioning and perspectives on the role and mandate for nurses in emergencies, crises, and disasters; (3) the role and responsibilities of nurses within the framework of public health‐related civil protection; and (4) education and training of nursing staff in German‐speaking countries to ensure targeted preparedness for emergencies, crises, and disasters.

For example, participants were asked to assess the importance of legally defining the role and mandate of nurses in such contexts, and to what extent they agreed that nurses in German‐speaking countries are generally capable of assuming safety‐critical roles during disasters. Alongside Likert scales, participants were invited to provide open‐ended comments on their responses to contribute additional insights they considered relevant.

Participation in the first online survey was limited to the round‐table attendees, ensuring that the outcomes of the symposium could be summarized, prioritized, and consolidated effectively. The next two rounds of the online survey – corresponding to the third and fourth stages of the Delphi process – were increasingly standardized. These subsequent rounds featured more specific questions, allowing for further refinement and primarily quantitative validation. The statistical results of the previous online survey rounds were presented graphically in the questionnaires of the follow‐up rounds (third and fourth Delphi rounds) in order to provide the experts with the feedback typical of Delphi studies (Cuhls 2023). Mean values were reported along with the confidence interval aggregated across all expert groups.

### Ethical Considerations

This study was conducted following the ethical principles outlined in the Declaration of Helsinki. Participation in the Delphi process was entirely voluntary, and written informed consent was obtained from all participants before their inclusion in the study. Participants were informed about the study's purpose, procedures, data protection measures, and their right to withdraw at any time without consequences. No personal identifying information was collected, and all data were anonymized before analysis. The study design did not involve vulnerable populations, clinical interventions, or the collection of sensitive health data.

Ethical approval for this study was obtained from the ethics committee of the Charité – Universitätsmedizin Berlin (Reference number EA4/087/24) on 29 May 2024.

### Data Analyses

The minutes of the round‐table symposium and the responses to open‐ended survey questions were analysed using the coding reliability form of thematic analysis following Braun and Clarke (2021), applying a semantic approach. Coding was conducted independently by two researchers with subsequent comparison and consolidation. Following data cleaning, quantitative data were descriptively analysed using the Statistical Package for the Social Sciences (SPSS), version 29. For the sample description, frequencies, means, and standard deviations were calculated depending on the scale level. In the presentation of Delphi results, mean values and standard deviations were used consistently across all items. For the third and fourth Delphi rounds, results were reported separately for nurses and those from other professional fields. Consensus was defined as mean agreement, with agreement assumed if the mean value exceeded five on a six‐point scale.

## Results

### Significance of the Topic

Participants were first asked to assess the importance of discussing, defining, and legally codifying the role and mandate of nurses in disasters. Across all three Delphi rounds, the topic was consistently rated as highly relevant. Participants also rated the necessity of reaching a broad societal consensus and establishing a legally binding framework for the role of nurses as highly significant. While both groups of experts, i.e., those who were nurses themselves and those without this qualification, emphasized the urgency of this policy issue, the nurses tended to assign slightly greater importance, particularly about the need for a clear regulatory definition of nurses’ role in disaster contexts.

### Role and Mandate of Nurses in Emergencies, Crises, and Disasters

The second section of the questionnaire addressed the safety‐critical role of nurses, including the perceived capacity to fulfil such roles and the conditions required to support this. There was strong agreement that nursing constitutes a fundamentally safety‐critical profession within the healthcare systems of the German‐speaking countries. This perception was more pronounced among nurses, while responses from experts outside the nursing profession were more varied. Additionally, participants suggested that nurses themselves may not consistently perceive their safety‐critical role.

When asked to prioritize structural prerequisites for enabling nurses in disaster contexts, participants ranked five predefined factors: financial resources, human resources, involvement in decision‐making bodies, professional autonomy, and systematic, accredited education. Across groups and rounds, financial resources were consistently assigned a lower priority. In contrast, involvement of (disaster) nurses in decision‐making bodies emerged as the top priority among experts who hold a licence in nursing. Experts with other professional backgrounds generally ranked this factor slightly lower, in third or fourth place out of the five factors.

### Role and Mandate of Nurses in the Context of Public Health Protection

Participants were also asked to evaluate statements concerning the role and mandate of nurses in public health protection. There was broad agreement on the importance of appointing disaster representatives within health care institutions and on strengthening the resilience of long‐term care settings. However, nurses emphasized more strongly the formal inclusion of experienced nurses in crisis management teams. Participants agreed that nurses have a special responsibility for vulnerable populations during disasters – though again, this was more strongly affirmed by nurses themselves.

### Education of Nurses for Targeted Preparation for Disasters in German‐Speaking Countries

The final section focused on education and training in disaster nursing, with the findings revealing an agreement on shortcomings in the current nursing education systems concerning emergencies, crises, and disasters. Nurses, however, expressed a stronger need for targeted educational reforms and the development of specific teaching materials.

Both groups strongly supported the need for further education for disaster representatives and the establishment of interprofessional educational programmes, although nurses rated the urgency of these measures even higher.

The ranking of educational measures included the following predefined options: conducting interprofessional disaster response exercises, implementation and adaptation of legal frameworks, teaching ICN competencies in pre‐licensed nursing education, and teaching ICN competencies in continuing education and training. Among these, teaching ICN competencies in basic, general nursing education consistently achieved the top position across both groups. This was followed by the implementation and adaptation of legal frameworks and the conducting of interprofessional disaster response exercises. Continuing education and training were also regarded as important, but ranked lower compared to measures aimed at pre‐licensed nursing education.

Table 3 provides a detailed overview of the Delphi findings across all thematic areas, including differences between nurse participants and other participants.

**TABLE 3:** Results of the second, third, and fourth Delphi stages.

Statement	First online survey (Delphi stage 2)	Second online survey (Delphi stage 3)		Third online survey (Delphi stage 4)	
		Nurses	Others	Nurses	Others
*Significance of the topic*	*M (SD)*	*M (SD)*	*M (SD)*	*M (SD)*	*M (SD)*
I believe that discussing the role and mandate of nurses in emergencies, crises, and disasters is …^a^	5.76 (0.54)	5.74 (0.71)	5.55 (0.64)	5.87 (0.35)	5.56 (0.66)
I believe that clearly defining the role and mandate of nurses in emergencies, crises, and disasters by law is…^a^	5.55 (0.68)	5.39 (1.09)	5.07 (0.94)	5.23 (0.90)	5.03 (0.94)
I believe that reaching a social consensus on the role and mandate of nurses in emergencies, crises, and disasters is …^a^	5.33 (0.97)	5.41 (0.98)	4.89 (0.96)	5.37 (0.81)	4.88 (1.04)
*The role and mandate of nurses in disasters, crises, and emergencies*	*M (SD)*	*M (SD)*	*M (SD)*	*M (SD)*	*M (SD)*
In my opinion, the healthcare profession of nursing is …^b^	95.9 (7.6)	96.9 (5.9)	86.3 (20.6)	95.2 (7.5)	89.4 (16.2)
Nurses probably perceive their profession as …^b^	60.3 (30.0)	68.6 (24.1)	70.3 (21.3)	68.0 (20.7)	69.4 (26.5)
Nurses in German‐speaking countries are basically able to take on safety‐critical roles in emergencies, crises, and disasters.^c^	3.76 (1.48)	4.22 (1.26)	3.89 (1.23)	4.07 (0.94)	4.26 (1.21)
*The role and mandate of nurses in the context of public health protection*	*M (SD)*	*M (SD)*	*M (SD)*	*M (SD)*	*M (SD)*
In all healthcare facilities where persons in need of care are accommodated and cared for, emergency, crisis, and disaster representatives from the nursing staff must be appointed.^c^	5.71 (0.56)	5.70 (0.66)	5.29 (1.27)	5.73 (0.52)	5.53 (0.71)
The resilience of long‐term care facilities (including home care) to emergencies, crises, and disasters must be systematically enhanced.^c^	5.55 (0.83)	5.61 (0.86)	5.71 (0.54)	5.77 (0.50)	5.71 (0.52)
Nurses with special expertise and experience in dealing with emergencies, crises, and disasters should also be regularly involved in crisis management teams.^c^	5.76 (0.54)	5.84 (0.52)	4.89 (1.57)	5.77 (0.77)	5.24 (1.10)
Nurses can play an active role in providing health information to selected population groups with regard to emergencies, crises, and disasters.^c^	5.43 (0.75)	5.59 (0.83)	5.36 (0.73)	5.83 (0.38)	5.35 (0.69)
Nurses assume special responsibility for the safety of population groups with special needs (e.g., the elderly, people in need of care, technology‐dependent people, children).^c^	5.71 (0.56)	5.74 (0.65)	5.33 (0.92)	5.83 (0.38)	5.47 (0.90)
*Nurses’ disaster education and training*	*M (SD)*	*M (SD)*	*M (SD)*	*M (SD)*	*M (SD)*
In German‐speaking countries, nurses are prepared by their education to perform their tasks in emergencies, crises, and disasters.^c^	2.24 (1.18)	2.26 (1.02)	2.54 (0.95)	2.27 (0.87)	2.59 (1.32)
There should be regular further education opportunities for nurses who take on tasks as disaster representatives (in care facilities).^c^	5.71 (0.56)	5.91 (0.29)	5.52 (1.04)	5.80 (0.41)	5.71 (0.63)
In order to prepare nurses for their tasks in emergencies, crises, and disasters, specific concepts, curricula, as well as learning and teaching materials need to be developed.^c^	5.62 (0.67)	5.83 (0.38)	5.25 (1.43)	5.77 (0.68)	5.56 (0.75)
There should be interprofessional education programmes in theory and practice for the health professions (including nurses) and disaster relief workers, as well as professionals in public emergency services.^c^	5.67 (0.73)	5.76 (0.57)	5.39 (1.34)	5.83 (0.38)	5.47 (0.83)

^a^ 1 = … not important at all, 6 = … very important.

^b^ 0 = … not a safety‐critical profession, 100 = … a safety‐critical profession.

^c^ 1 = strongly disagree, 6 = strongly agree.

## Discussion

This Delphi study revealed a clear consensus among experts on the need to strengthen and codify the role and mandate of nurses in disaster contexts across German‐speaking countries. While international frameworks increasingly emphasize the relevance of nurses in disaster contexts, Germany, Austria, and Switzerland lack the necessary policy, legal, and educational foundations. Above all, there is no structured debate led by nurses on the role and mission of the profession, not only but especially in disasters.

The process of clarifying roles and mandates regarding disasters and public health must begin within the nursing profession itself. This is crucial to prevent others from doing it in ways that weaken or undermine the profession's autonomy and position (White et al. 2025). It is therefore all the more worrying that the findings of our Delphi study suggest that experts believe that nurses themselves may not always be aware of their safety‐critical role in such contexts or may not always accept it. This could be due to the historically subordinate role of nurses in healthcare and health policy, limited political engagement, and insufficient academic or organizational capacities within the profession in German‐speaking countries. Although there are now louder calls for professional autonomy and self‐determination in nursing in these countries, the greater responsibility that this entails is not always accepted. This is likely to significantly hinder the further development of profession‐specific agendas, particularly about the role and mandate of nurses in disaster situations.

To change this, a broad opinion‐forming debate is needed within the profession and its professional organizations. The new ICN's definitions of nursing and nurse provide important reference points on the profession's response to disasters (White et al. 2025). However, it will take time to implement these definitions in national discourse and practice in German‐speaking countries. At present, it is still uncertain to what extent nurses there will adopt these definitions and adapt to their implications. However, it is unlikely that other stakeholders will advocate for these claims on behalf of nurses, as is also suggested to some extent by the results of this Delphi study. Participants who are not nurses consider clarifying the role and mandate of nurses in disasters to be less important than nurses themselves. Therefore, as Avraham et al. noted, nurses themselves “should take an active role, advocate for progress, and assume responsibility for the development of their profession” (Avraham et al. 2025, p. 9) to bring about much‐needed change.

Once nurses and their professional organizations have clarified and defined their role and mandate in general and particularly about disasters, this must be communicated to political decision‐makers to recognize, consolidate, and codify this professional self‐conception in a legal and regulatory framework. Delphi participants, especially nurses, strongly supported the formal anchoring of nurses’ roles in disaster contexts through clearly defined mandates and structural integration in decision‐making bodies of disaster management. However, there has been virtually no political engagement with the demands of nurses – particularly those concerning mandates for population protection, professional leadership, and educational reform, as emphasized in this study. Accordingly, the codification of binding national legislation that would define nurses’ scope of practice, their responsibilities, and leadership roles remains the exception in German‐speaking countries.

Also, internationally, there is a need to legally define the mandate of nurses in disasters, especially to give them legal protection (Avraham et al. 2025). After centuries of building legitimacy and ongoing under‐recognition alongside other professions, disaster nursing is only now gradually gaining public recognition (Fletcher et al. 2022). This recognition stems from the holistic perspective of nurses and therefore a community focus (Springer 2018), which is less included in other professions but highly important in disaster preparation and response. However, here too, nurses’ political invisibility and, above all, their political marginalization, as highlighted by Rasheed et al. (2020), could continue to hinder the legislation of nurse‐driven political initiatives in this area. This marginalization of nurses, and especially nurse policymakers, must be overcome so that their role is strengthened and their opinions are heard and valued (Rasheed et al. 2020). Because without legal codification, nurses’ role and mandate in disaster contexts risk remaining vague and institutionally unanchored – limiting not only their operational capacity in exceptional situations but also their professional and strategic influence in shaping policies on prevention and reduction of disaster risks.

Not least, for systems to eventually become truly resilient and capable of responding to disasters, the role and mandate of nurses must not only be defined and recognized but also embedded within the structures, routines, and cultures of disaster preparedness and response (Avraham et al. 2025). This implementation, however, requires political will, sustained funding, intersectoral cooperation, and a commitment to supporting nursing leadership across all levels of disaster governance. Delphi participants emphasized the importance of such structural integration of nurses, particularly through formal roles in decision‐making bodies. This perspective was especially strong among nurses themselves, but also found support across professional boundaries.

It should be noted at this point that there are still considerable gaps internationally in the consistent integration of experienced nurse leaders into disaster management structures (NASEM 2021; Veenema et al. 2016), with educational aspects often discussed as a crucial factor (Veenema et al. 2017). This might be particularly justified in German‐speaking countries. The Delphi findings showed broad agreement that current nursing education does not adequately prepare nurses in these countries for the specific demands of disaster preparedness and response. Disaster‐related content is largely absent from basic education, and opportunities for advanced training are limited. This underscores the urgent need to make essential disaster nursing competencies a mandatory part of the curriculum and legal frameworks for nurse education in German‐speaking countries. In addition, professional development options at an advanced practice level should be offered on this topic in order to provide the necessary leadership resources. The first steps in this direction have already been taken with the authorized translation and cultural adaptation of the ICN Core Competencies for German‐speaking countries (Ewers and Köhler 2025). However, this must be followed by further concerted action in the area of education and training.

But to really get nurses involved in disaster preparedness and response structures, their aforementioned structural marginalization and lack of visibility need to be addressed. Nurses, not only in the German‐speaking countries but worldwide, must be empowered to raise their voices, take responsibility and leadership roles within disaster management structures, and take advantage of opportunities to shape institutional policy (Rasheed et al. 2020). Simultaneously, health and disaster relief authorities and institutions must evolve to provide the necessary space for such participation – both through the formal creation of disaster nursing roles and a cultural shift towards recognizing nurses as strategic actors and partners.

### Strengths and Limitations

This Delphi study is the first to address the role and mandate of nurses in disasters within the German‐speaking region. The results can potentially be used as a valuable basis for further national policy discussions and may enrich international debates on the role of nurses in disaster preparedness and response structures. Although every effort has been made to avoid limitations, the following points should be carefully considered when interpreting the results.

First, the Delphi sample was non‐representative and composed of participants selected for their subject‐specific expertise. This approach likely limited the inclusion of broader, potentially dissenting perspectives. Although iterative expansion of the panel across survey rounds was intended to capture a wider range of perspectives, the sample's composition still represents a key limitation in the generalizability and comprehensiveness of the findings.

Second, the panel size remained within an acceptable range for Delphi studies. Nevertheless, the relatively low response rate – especially in the final two rounds – may have introduced bias towards more engaged or interested stakeholders.

Third, the representation of participants from the three German‐speaking countries (Germany, Austria, and Switzerland) may also have been uneven. Although invitations were distributed proportionally across all three countries, the final responses cannot be disaggregated by country due to anonymization.

Fourth, the findings are closely tied to the structural, educational, and political context of the German‐speaking countries. Even if the findings cannot be directly transferred to other national contexts – especially those with different healthcare systems, roles of nursing staff, or disaster management structures – the insights gained here can serve as a starting point for further discussion.

## Conclusion

This Delphi study in the German‐speaking countries draws attention to the urgent need to define, strengthen, and structurally anchor the role and mandate of nurses in disaster preparedness, response, and recovery within this region. This is particularly salient given that the enhancement of the nurses’ role in disasters is not merely a matter of professional advancement; rather, it represents a strategic necessity that may also be relevant from an international perspective for the development of resilient, equitable, and responsive health systems in times of disasters.

In that sense, the results of this Delphi study can inform nursing policy both within the profession and beyond. They can be understood as a call for action to engage more intensively with the topic – not only among nurses themselves, but also across political and societal arenas. This includes initiating a broader public discourse within the German‐speaking countries and possibly beyond about what society expects from nurses and nursing, and what responsibilities and leadership it wants to assign to this profession. Further surveys – including those of members of nursing colleges, nursing associations, and organizations nationally and internationally – could provide additional insights into nurses’ self‐perception and help to clarify the role and mandate of nursing from within.

### Implications for Nursing and Health Policy

The findings of this Delphi study provide a strong foundation for advancing nursing policy, both within the profession and in broader health governance and institutions. This requires a strategic realignment. First, the nurses themselves must define and communicate their role in disaster prevention and management. Political decision‐makers must then translate this into legally binding codified mandates, and finally, all relevant actors and institutions must do everything in their power to integrate this role and the mandate of nurses into disaster risk management structures. The definition, codification, and implementation of disaster management capacities are essential to ensure an inclusive and robust response to increasingly complex disasters and to ensure public safety. Therefore, political leadership – within and beyond the nursing profession – and a coordinated roadmap are needed to harness the potential of nurses for disaster resilience.

## Author Contributions

Study design: MK and ME. Data collection: MK, JB, and LG. Data analysis: MK and JB. Study supervision: ME. Manuscript writing: MK, JB, and ME. Critical revisions for important intellectual content: MK, JB, LG, and ME.

## Conflicts of Interest

No conflict of interest has been declared by the authors.

## Funding

The authors have nothing to report.
